# Improvement of Single-Crystal Structures of Very Heavy Element Compounds by Refining Anomalous Dispersion Parameters

**DOI:** 10.1021/acs.inorgchem.4c01772

**Published:** 2024-08-08

**Authors:** Florian Meurer, Gregory Morrison, Birgit Hischa, Hans-Conrad zur Loye, Christoph Hennig, Michael Bodensteiner

**Affiliations:** † Faculty for Chemistry and Pharmacy, University of Regensburg, Universitätsstrasse 31, Regensburg 93053, Germany; ‡ Institute of Resource Ecology, Helmholtz-Zentrum Dresden-Rossendorf (HZDR), Bautzner Landstrasse 400, Dresden 01314, Germany; § Department of Chemistry and Biochemistry, University of South Carolina, Columbia, South Carolina 29208, United States; ∥ Rossendorf Beamline (BM20-CRG), European Synchrotron Radiation Facility (ESRF), 71, Avenue des Martyrs, Grenoble 38043, France

## Abstract

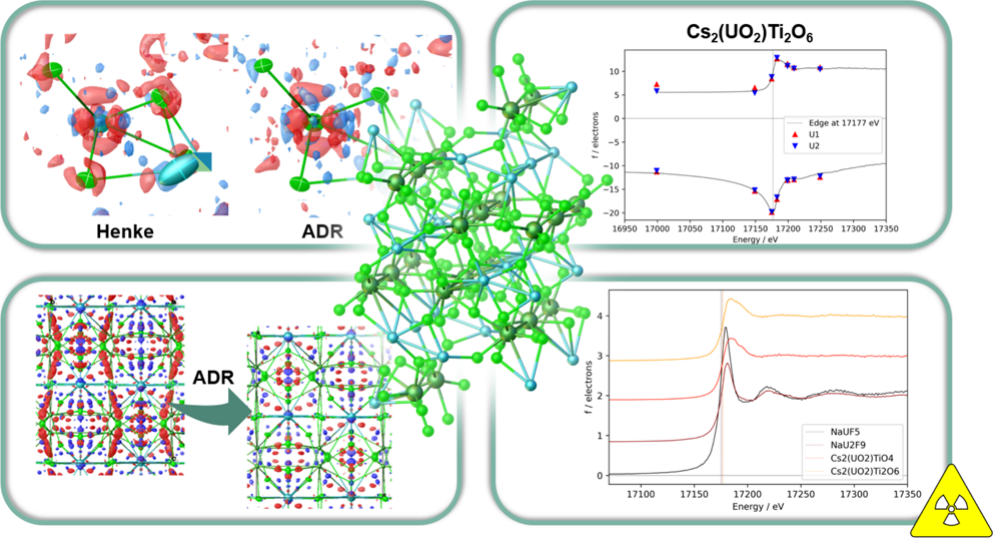

Refining the anomalous dispersion parameters of the four uranium compounds NaUF_5_, NaU_2_F_9_, Cs_2_(UO_2_)TiO_4_, and Cs_2_(UO_2_)Ti_2_O_6_ gave insights into the crystallographic model improvement of very heavy atoms. We found that the values for the dispersive and absorptive parts, *f*′ and *f*″, closely followed the X-ray absorption spectra on their L_3_, L_2_, and L_1_ edges. The obtained values are sensitive to the chemical environment at each crystallographically independent position. An incorrect treatment of the anomalous dispersion correction can lead to a wrong crystallographic model. The above-mentioned, already published structures were improved by this process. General guidelines were given for the crystal structure determination of very heavy compounds. When using Mo Kα radiation with uranium compounds, the proximity of its energy to the uranium L-edges causes a noticeable effect.

## Abstract 2

The refinement of the anomalous dispersion (ADR) correction parameters is tested for very heavy atomic single-crystal data using four uranium compounds as examples. ADR was capable of closely following the X-ray absorption spectrum of each compound along all L absorption edges of uranium. Additionally, chemically equivalent, but crystallographically inequivalent uranium sites showed chemical sensitivity toward the spatial position.

## Introduction

In single-crystal X-ray diffraction (SC-XRD), the anomalous dispersion terms *f*′ and *f*″ correct for the nonelastic behavior of a given element. They correspond directly to an X-ray absorption spectrum (XAS) and its Kramers–Kronig transformation (KKT), which contain valuable chemical information when recorded over several energies near the absorption edge of an element.^1,2^ In protein crystallography, this relation has been shown to provide valuable spatially resolved chemical information in important systems such as nitrogenase FeMoco and the FeV cofactor.^3−5^ It has received much less attention for small-molecule SC-XRD, with rare examples being presented, for instance, by Bartholomew et al.^6,7^ This is surprising as the initial research into the refinement of the anomalous dispersion correction terms goes back as early as 1978 and was well examined by L. K. Templeton and D. H. Templeton.^8,9^

In 2022, we have described the application and implementation of anomalous dispersion refinement (ADR) within one of the most widely used crystallographic software suites Olex2.^10^ In this work, a good agreement was found between a recorded X-ray absorption spectrum (XAS) of the organometallic compound Mo(CO)_6_ and its KKT with the refined values for *f*″ and *f*′ obtained directly from the diffraction data.

Most recently, Leinders et al. published their investigations on ADR of tetravalent uranium dioxide as well as pentavalent KUO_3_ with powder diffraction.^11^ In this study, the dispersive part of the atomic form factor *f*′ was obtained by the transformation of refined *f*″ values and introduced as a parameter in the least-squares refinement procedure. In contrast, we refined both *f*′ and *f*″ for uranium directly from the experimental intensities from single-crystal data.

Uranium-containing compounds are interesting both chemically and crystallographically. Their chemistry has been and continues to be extensively studied, especially in the context of nuclear waste deposits.^12^ The different oxidation states and bonding motifs of uranium make it interesting from a crystal chemistry viewpoint. In oxides, the +6 oxidation state is most prominent, and U(VI) almost always forms the uranyl cation, UO_2_^2+^, which consists of a central uranium atom strongly bound to two oxygens with an almost 180° arrangement. The uranyl uranium is further coordinated by four, five, or six equatorial oxygens to form square, pentagonal, or hexagonal bipyramids.^13^ The two strongly coordinated uranyl oxygens rarely participate in further bonding^14^ except to weakly bond to low valent cations such as the alkali cations.^15,16^ For this reason, the uranyl ion promotes the formation of layered and channel structures. In fluorides, uranium often adopts the +4 oxidation state and typically forms irregular 8- or 9-coordinate polyhedra.^13,17,18^

Crystallographically, uranium typically exhibits a large residual electron density in its vicinity, a common problem with very heavy elements. Particularly for these elements, the values for anomalous dispersion correction can become quite large. For example, when uranium compounds are measured using Mo Kα radiation (*E* = 17.4 keV), just above the uranium L_3_ absorption edge (*E* = 17.1 keV), a significant fraction of the incoming radiation is absorbed and dispersed (*f*′ = −10.2 e, *f*″ = 10.4 e according to Henke et al.^19^). As the atomic scattering power is closely connected to the atomic position, displacement parameters, and most notably the element type, an adequate correction for these effects is essential for determining the correct structure. The quality of the structural model strongly depends on the treatment of these effects.

Herein, we present the capability and robustness of anomalous dispersion refinements (ADR) carried out at the L absorption edges of the known uranium single-crystalline compounds NaUF_5_, NaU_2_F_9_, Cs_2_(UO_2_)TiO_4_, and Cs_2_(UO_2_)Ti_2_O_6_.^18,20^ ADR was able to capture the features within the strongly pronounced L_3_ edge of these compounds as well as the weaker L_2_ and L_1_ edges. For NaUF_5_, we present the dependency of ADR at room temperature (RT) as well as at 100 K. As ADR introduces two additional variables per resonating atom to the crystallographic model, it is important to investigate to what extent ADR improves the residual electron density and whether the correction parameters compensate for other effects.

## Methods

To investigate the behavior of ADR at heavy element L-edges, SC-XRD experiments were performed at various energies around the uranium L-edges (L_3_: 17.166 keV, L_2_: 20.948 keV, L_1_: 21.757 keV^21^). Simultaneously, X-ray absorption spectra were recorded on the same single crystals in fluorescence mode in the range of these energies. All experiments were performed at the Rossendorf Beamline (BM20) at the European Synchrotron (ESRF) in Grenoble, France.^22^

X-ray diffraction data were processed in CrysAlisPro^23^ and evaluated in Olex2^24^ using olex2.refine^25^ as a refinement engine employing the serial processing module SISYPHOS.^26^ All data were treated with only multiscan absorption correction^27^ to avoid including the linear absorption coefficient μ. This is due to the direct proportionality between μ and *f*″, the absorbing part of the dispersion correction. Especially in the edge region, the tabulated values are the most incorrect, as both the edge position and the fine structure features are individuals for each substance. Therefore, any absorption correction based on μ is also incorrect, particularly in this area.

XAS data were processed using the PyMCA^28^ software suite, and the Kramers–Kronig transformations were performed using the kkcalc^29^ program. Further details can be found in the Supporting Information (SI).

## Results and Discussion

**Figure 1:**
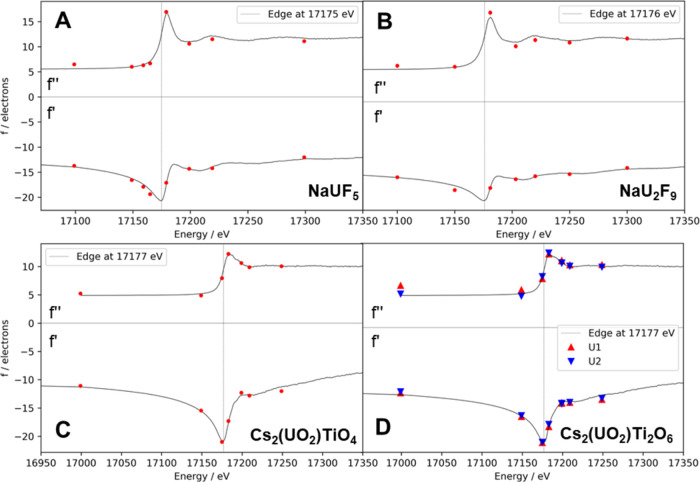
Each plot shows the recorded XAS (top) and its KKT (bottom) for the L_3_ edge of uranium in four different compounds, together with the freely refined values for *f*″ and *f*′ from SC-XRD data at different energies. Absorption edges, determined from the strongest maximum in the first derivative of the XAS, are also given (vertical gray lines). The XAS and its KKT were first normalized and then fitted to the ADR parameters.

The results from ADR together with the recorded U L_3_ edge XAS data in both uranium(IV) fluorides and uranyl titanates are shown in Figure 1. In all cases, we observed good agreement between the XAS/KKT and the independently refined values for *f*″/*f*′ obtained from SC-XRD data. The absolute values for *f*′ and *f*″ follow the fine structure in the near edge region of XAS. The two crystallographically independent positions for uranium in Cs_2_(UO_2_)Ti_2_O_6_ (Figure 1D) are chemically equivalent. This was reflected by very similar ADR values and underlined the sensitivity of ADR toward chemical features.

**Figure 2:**
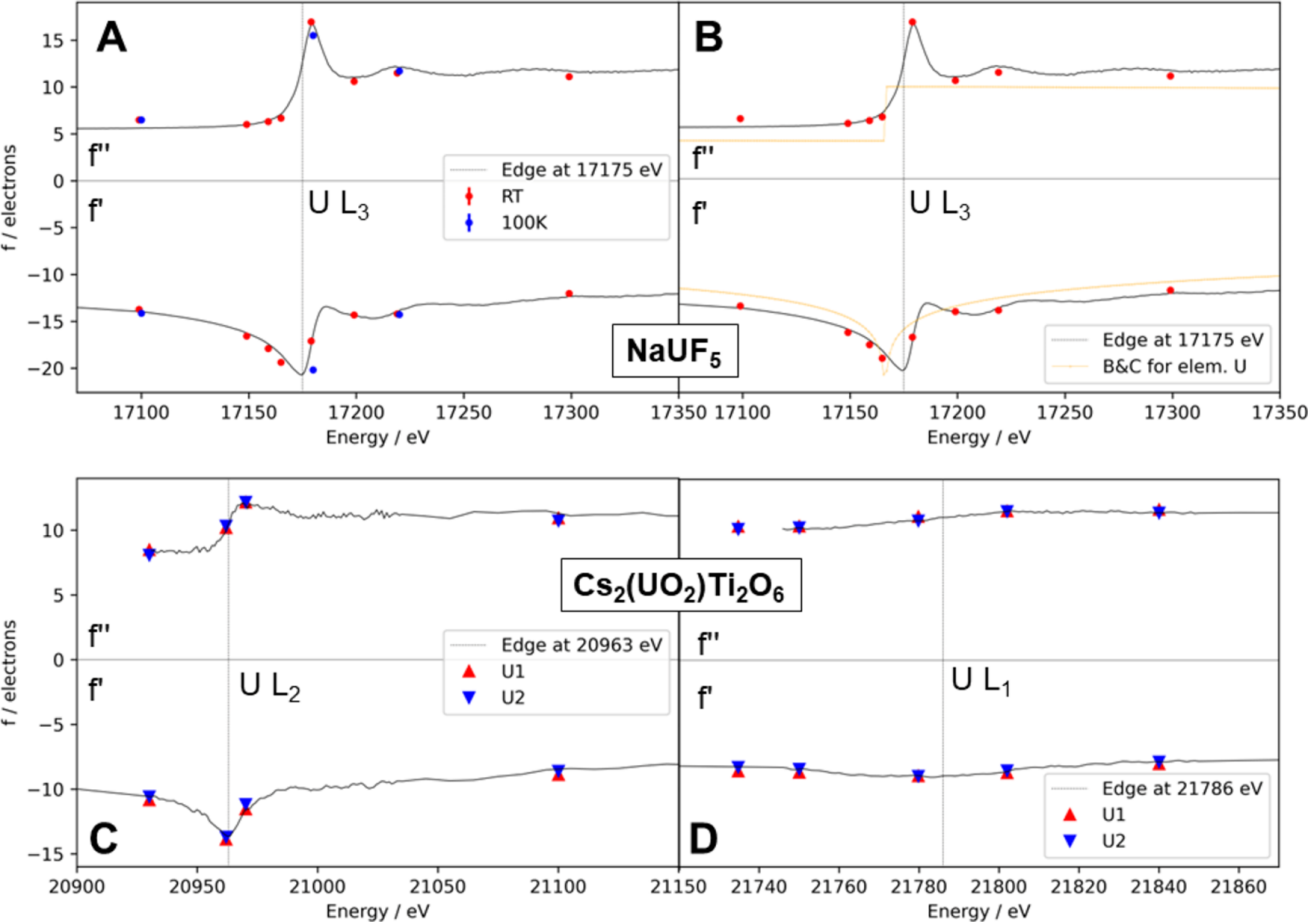
Each plot shows the recorded XAS (top) and its KKT (bottom) for NaUF_5_ (A, B) and Cs_2_(UO_2_)Ti_2_O_6_ (C, D), together with the freely refined values for *f*″ (top) and *f*′ (bottom) from XRD data at the different energies. A shows the ADR parameters at room temperature (red) as well as at 100 K (blue). (B) shows the values compared to the calculated values for uranium according to Brennan and Cowan.^30^ Panels (C) and (D) compare the spectra to ADR values at the U L_2_ and U L_1_ absorption edge, respectively. The XAS and its KKT were first normalized and then fitted to the ADR parameters.

Figure 2 shows a good agreement between *f*′ and *f*″ and no significant temperature dependence for ADR of NaUF_5_ at two temperatures (RT and 100 K, A). Only at the edge energy, a deviation was observed. However, this difference could be due to a small energy difference that can occur when the synchrotron beam is readjusted. For technical reasons, the low-temperature measurements were not carried out directly after the room-temperature experiments. Especially in the absorption edge, such a rather small drift in energy can already lead to a considerable deviation in absorption.

Most prominently, a large difference was observed between the refined *f*′ and *f*″ values compared to the calculated dispersion correction according to Brennan and Cowan (Figure 2, B).^30^ Not only was there a typical offset due to the difference in edge energy between elemental uranium and uranium embedded in a chemical environment (here by 9 eV), but there was also a shift to higher absolute values for *f*″ and *f*′. This trend was also observed for the other compounds of this study (see Figure S4 in the SI). Furthermore, a good agreement was also found at the less pronounced L_2_ and L_1_ edges in the respective spectra and even for the two uranium positions in the case of Cs_2_(UO_2_)Ti_2_O_6_.

**Figure 3:**
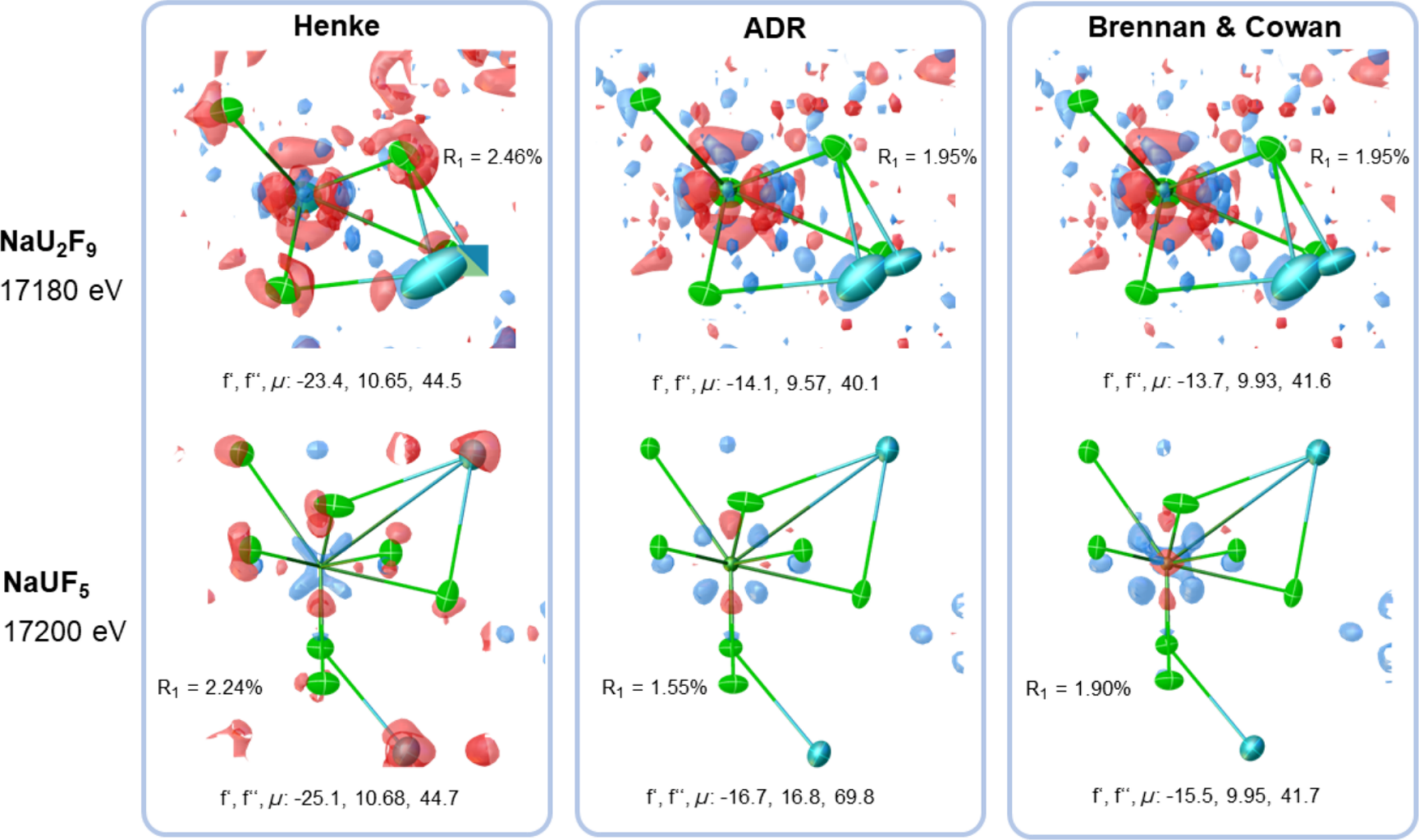
Residual electron density maps (blue: positive, red: negative) for the asymmetric units of NaU_2_F_9_ (top, 1.0 e·Å^–3^ iso-surface) and NaUF_5_ (bottom, 0.9 e·Å^–3^ iso-surface) modeled according to Henke^19^ (left, linearly interpolated), ADR (middle), and Brennan and Cowan^30^ (right, calculated). Ellipsoids are shown at 50% probability level, and tetrahedra indicate nonpositive definite displacement parameters. *f*′ and *f*″ values are given in electrons and elemental μ in kilobarns per atom.

In Figure 3, the residual electron density maps show the crystallographic models for three different sources of anomalous dispersion corrections above the absorption edge of uranium. In the case of NaU_2_F_9_, a significant difference between the model relying on the Henke table^19^ and the one for Brennan and Cowan^30^ was apparent. Using Henke the agreement strongly decreased and a disorder in the sodium position, far away from the uranium atom, could not be modeled properly. The values obtained from ADR closely matched those of Brennan and Cowan,^30^ and similar agreement was obtained between measured and modeled structure factors as indicated by the crystallographic *R*_1_ value.

In the case of NaUF_5_, ADR showed a better agreement with the measured data compared to the models using the values from Henke^19^ and Brennan and Cowan.^30^ This suggests that ADR provided the most accurate values for anomalous dispersion correction in this approach, especially where pronounced spectral features dominate the XAS.

Generally, Brennan and Cowan^30^ gave a good approximation, except for the edge region. This was particularly relevant for uranium compounds measured with Mo Kα radiation (*E*_Mo Kα_ = 17.4 keV), which lies just above the U L_3_ edge. This was reflected very well in the work of Gianopoulos et al.,^31^ where in a charge density study of uranium, the anomalous dispersion values refined very close to those calculated by Brennan and Cowan.^30^

Note that the resulting elemental absorption coefficient for uranium differed by more than 50% (69.8 kilobarns per atom with ADR, 41.7 kilobarns per atom according to Brennan and Cowan^30^). Such a difference can have a significant effect on the analytical absorption correction, which is essential for atomic structures containing heavy elements. Since *f*″ and μ are directly proportional to each other, their dependence is obvious. However, μ is used to correct the data, while *f*″ and *f*′ are used to correct the model. Therefore, the true value of μ could potentially be found by repeatedly refining *f*″ and then applying the resulting μ to the absorption correction until convergence is reached.

Using the respective charged atomic form factors for uranium (U^4+^ for the fluorides and U^6+^ for the titanates) showed only a little deviation from the values obtained by using the neutral uranium atomic form factor. While the fluorides showed no significant difference, the *f*′ obtained using charged uranium for the titanates did show lower absolute values than when using the neutral atomic form factor. This is consistent with the higher charge at the U^6+^ ion in the uranyl titanate structures. Again, the difference in *f*′ and *f*″ for the two uranium positions in Cs_2_(UO_2_)Ti_2_O_6_ was only marginal.

**Figure 4:**
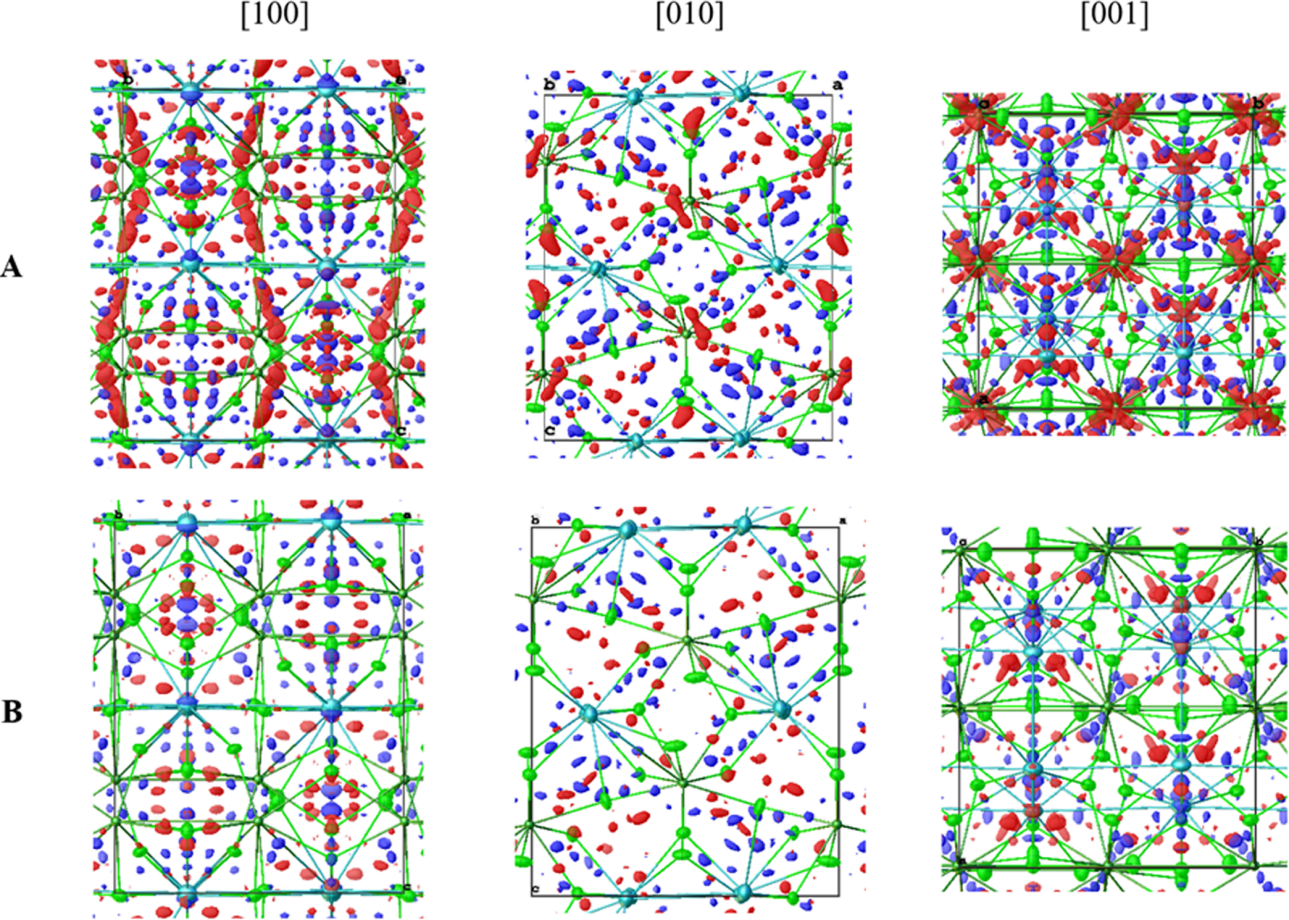
Residual electron density maps at 0.6 e·Å^–3^ iso-level (blue: positive, red: negative) for the structure of NaUF_5_ (CCDC 1827659)^18^ refined as published using Henke (A) and refined anomalous dispersion values (B) in crystallographic *a*, *b*, and *c* directions. Light green atoms: fluorine, dark green atoms: uranium, and cyan atoms: sodium.

As the structures of the four compounds in our study had previously been determined using Mo Kα radiation, we applied ADR to these previously published datasets.^18,20^Figure 4 shows the residual electron density maps of NaUF_5_ with and without refined anomalous dispersion correction parameters. This map describes the observed electron density, which could not be completely described by the crystallographic model. Therefore, we attributed the significant reduction of this density to the improved correction by ADR for the nonelastic behavior. Even though the published structure was already of high quality, ADR improved the agreement of observed and modeled intensities even more. Subsequently, both the maximum and the minimum residual electron density peaks decreased. Table 1 shows the respective improvements for all four compounds with their corresponding values for the anomalous dispersion correction.

**Table 1:** Refinement indicators achieved with the Brennan and Cowan^30^ (B&C) anomalous dispersion values as well as by ADR for the published data of the four compounds within this study^a^^,^^18,20^

	NaUF_5_		NaU_2_F_9_		Cs_2_(UO_2_)TiO_4_		Cs_2_(UO_2_)Ti_2_O_6_	
	B&C	ADR	B&C	ADR	B&C	ADR	B&C	ADR
*R*_1_, w*R*_2_/%	1.32, 2.64	1.26, 2.50	1.34, 3.07	1.33, 2.98	1.12, 2.52	1.08, 2.42	1.20, 2.87	1.08, 2.51
min., max. peaks/e·Å^–3^	–0.93, 1.07	–0.97, 0.97	–0.89, 1.11	–0.89, 1.03	–0.74, 0.73	–0.62, 0.67	–0.86, 1.67	–0.935, 1.53
weights	0.00, 2.91	0.00, 1.92	0.01, 0.87	0.01, 0.53	0.01, 21.0	0.00, 19.2	0.01, 11.7	0.01, 2.39
*f*′, *f*″/e	–9.7, 9.7	–11.9(3), 11.8(6)	–9.7, 9.7	–11.1(3), 9.9(6)	–9.7, 9.7	–10.2(4), 10.5(8)	–9.7, 9.7	–10.5, −11.3, 10.2, 10.1

a The published structures were re-refined using olex2.refine^25^ employing the recently published improved spherical model according to Thakkar.^32,33^ Additionally, for the uranyl titanate structures, the cesium atoms were refined anharmonically. Quality parameters were calculated to a resolution of 0.75 Å, and *f*′ and *f*″ were refined at the maximum resolution of the dataset.

Generally, ADR improved the crystallographic model, resulting in better quality parameters, lower residuals, as well as lower weights on both the low- and high-resolution reflections. The deviation from the tabulated anomalous dispersion parameters according to Brennan and Cowan^30^ (−9.7 e, 9.7 e) was rather large. The values by Brennan and Cowan^30^ differ more strongly from those of Sasaki^34^ (−11.0 e, 9.7 e) and Henke et al.^19^ (−10.2 e, 10.4 e). In particular, *f*″ reached higher values in the free refinement than all tabulated sources, especially prominent for NaUF_5_ with a value of 11.8 e.

Remeasurement of the crystals subject to this study on an in-house diffractometer (Rigaku XtaLAB Synergy-DW, HyPix-Arc 150°) generally showed good agreement compared to the published structures. Further information can be found in the SI.

We are therefore confident in recommending ADR for uranium structures measured using in-house diffractometers and Mo Kα radiation. However, this method should only be applied to high-quality datasets, without unresolved other issues, such as disorder, twinning, or anharmonicity. The warnings implemented in *Olex2* should always be carefully considered if the values resulting from the ADR differ significantly from tabulated values.

## Conclusions

In this work, we demonstrated the ability to perform anomalous dispersion refinements for very heavy elements in the energy range of their L absorption edges using four different uranium compounds as examples. The ADR′s *f*″ and *f*′ followed the independently recorded X-ray absorption spectra and Kramers–Kronig transformation closely, even in the delicate edge region. In the case of Cs_2_(UO_2_)Ti_2_O_6_, which contains two crystallographically independent but chemically identical uranium positions, the deviation of *f*′ and *f*″ for both positions was negligible. This underlines the benefit of refining individual dispersion parameters for spatially and chemically different elements. We have also shown that treatment of the anomalous dispersion as a free parameter leads to an improvement in the structural models of measurements with the laboratory diffractometer far away from the absorption edges. The published structures of the four compounds subject to this study, measured at an in-house diffractometer using common Mo Kα radiation, could be improved by applying ADR.

## Supporting Information Available

Descriptions of synthetic, crystallographic, and spectroscopic data (PDF)
The starting models for each of the four compounds are attached as a crystallographic information file (“.cif”); all results of the ADR procedure including the unedited.cifs as well as the tabulated results are given in ASM1 and ASM2; crystal structures using charged atomic form factors are given in ASM3; the details of the synthetic procedures and data processing as well as further crystallographic details; ASM1—containing tabulates results from the ADR procedure (.zip, containing.csv); ASM2—crystal structures from the ADR procedure (.zip, containing.cif); ASM3—crystal structures of charged atomic form factors (.zip, containing.cif) (ZIP)
