# Supplementary material for: Improvement of Single-Crystal Structures of Very Heavy Element Compounds by Refining Anomalous Dispersion Parameters
Source: Inorg Chem. 2024 Aug 8;63(34):15784–90. doi: 10.1021/acs.inorgchem.4c01772 (PMC11351177; doi:10.1021/acs.inorgchem.4c01772)
Supplement: Supplementary file 1 — ic4c01772_si_001.pdf [file ic4c01772_si_001.pdf]

Supporting Information

Improvement of Single-Crystal Structures of Very Heavy Element Compounds by Refining Anomalous Dispersion Parameters

Florian Meurer[a,b], Gregory Morrison[c], Birgit Hischa[a], Hans-Conrad zur Loye[c], Christoph Hennig[b,d] and Michael Bodensteiner[a]**

[a] Faculty for Chemistry and Pharmacy, University of Regensburg, Universitätsstrasse 31, Regensburg 93053, Germany

[b] Institute of Resource Ecology, Helmholtz-Zentrum Dresden-Rossendorf (HZDR), Bautzner Landstrasse 400, Dresden 01314, Germany

[c] Department of Chemistry and Biochemistry, University of South Carolina, Columbia, South Carolina 29208, United States

[d] Rossendorf Beamline (BM20-CRG), European Synchrotron Radiation Facility (ESRF), 71, Avenue des Martyrs, Grenoble 38043, France

Correspondence: michael.bodensteiner@ur.de, christoph.hennig@esrf.fr

Synthesis and General Considerations

Single crystals of the four compounds were prepared using the published experimental procedures.[1,2] Briefly, $NaUF_5$ and NaU_2F_9 were synthesized under mild hydrothermal conditions. For $NaUF_5$, 0.2500 g of UF_4, 0.0361 g of NaF, 0.25 mL of 49% HF, and 0.5 mL of distilled water were enclosed in an autoclave with a 23 mL PTFE liner. For NaU_2F_9, 0.1500 g of UF_4, 0.0108 g of NaF, 0.25 mL of 49% HF, and 1.5 mL of distilled water were enclosed in an autoclave with a 23 mL PTFE liner. Both reactions were heated to 200 ºC for 36 h in a programmable oven and then allowed to cool naturally by shutting off the oven. The crystals were isolated from the mother liquor via vacuum filtration and washed with water followed by acetone. $Cs_2(UO_2)TiO_4$ and $Cs_2(UO_2)Ti_2O_6$ were grown via flux growth from a near eutectic cesium fluoride/cesium chloride melt. For $Cs_2(UO_2)TiO_4$ and $Cs_2(UO_2)Ti_2O_6$, a mixture of 1/6 mmol U_3O_8 and ½ mmol TiO_2 and a mixture of 0.5 mmol UF_4 and 1 mmol TiO_2, respectively, were layered under a mixture of 9 mmol CsF and 11 mmol CsCl in a cylindrical silver crucible. The reactions were rapidly heated to 900 ºC for 12 h, cooled to 400 ºC at 6 ºC/h, and then allowed to cool naturally by shutting off the furnace. The flux was dissolved via sonication in water and the crystals were isolated via vacuum filtration and washed with water followed by acetone. The resulting crystals were analyzed on an in-house Bruker D8 Quest diffractometer equipped with a microfocus Mo Kα X-ray source and a Photon II detector to select high-quality crystals for subsequent analysis on the beamline.

In $Cs_2(UO_2)TiO_4$, and $Cs_2(UO_2)Ti_2O_6$ the uranium ions form uranyl ions and have UO_6 square bipyramidal coordination. In $Cs_2(UO_2)TiO_4$, these UO_6 polyhedra corner share through two equatorial oxygens to form chains, with the other two equatorial oxygens corner-sharing to TiO_4 tetrahedra. The -yl oxygens are terminal and stick into channels occupied by the Cs cations. The UO_6 polyhedra consist of two short -yl bonds with length 1.873(2) Å, two intermediate bonds along the chains with length 2.1459(4) Å, and two longer bonds of length 2.2431(18) Å.[2] $Cs_2(UO_2)Ti_2O_6$ has two unique uranium positions, both forming uranyl ions and having UO_6 square bipyramidal coordination. The bonding environments for the two uranium positions are very similar containing two -yl oxygens with bond distances of 1.834(3) – 1.837(3) Å (U1) and 1.831(2) Å (U2) and four equatorial oxygens that corner share with TiO5 polyhedra and have bond distances of 2.2177(18) – 2.2338(18) Å (U1) and 2.2115(19) – 2.2373(19) Å (U2).[2] $NaUF_5$ and NaU_2F_9 each have one unique uranium position, forming UF_9 polyhedra. The bond distances in $NaUF_5$ have a greater range, 2.134(2) – 2.486(2) Å, compared to those in NaU_2F_9, 2.24775(17) – 2.418(2) Å.[1]

Experimental Procedures

All data were obtained at the Rossendorf Beamline (ROBL, BM-20) at the European Synchrotron (ESRF) in Grenoble, France.[3]

X-ray absorption spectroscopy data for the comparison spectra were recorded in fluorescence mode using a FalconX single-module ROI-based fluorescence detector. The energy was calibrated against the first inflection of the first derivative for an Y foil at 17.038,0 eV (see Fig. S1).

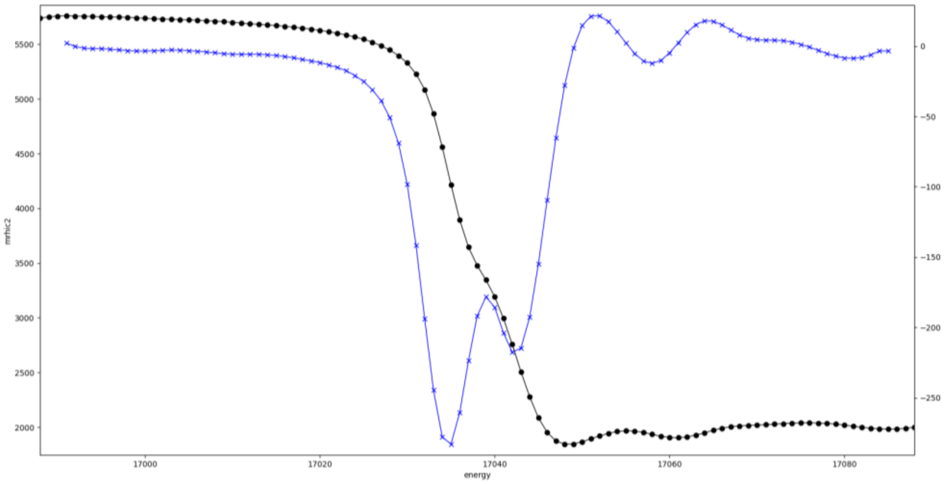

Figure S1: Absorption spectrum (transmission mode) for an Y foil for energy calibration. The minimum of the derivative was found at 17,035 eV and then set to the K absorption edge of Y at 17,038 eV.

The absorption spectra at the uranium L_3-edge are shown in Fig. S2.

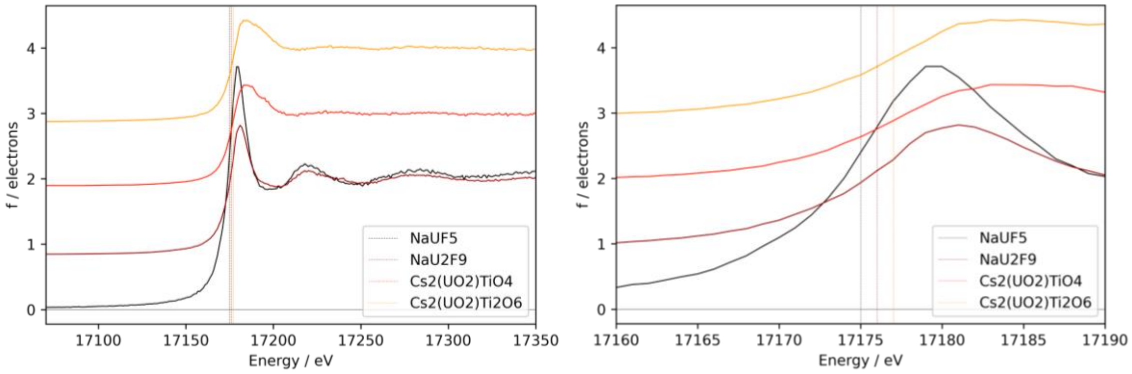

Figure S2: Absorption spectra in fluorescence more for the compounds of this study.

Diffraction data were collected on a Huber Kappa diffractometer using a DECTRIS PILATUS3 X 2M photon counting detector for recording data. The data were processed using the SNBL toolbox[4] and CrysAlisPro[5]. The instrument model was calibrated using an LaB_6 powder standard using *PyFAI*[6] and the resulting instrument model was refined against an "Ylid"[7] single-crystal dataset with 100.0% of recorded reflections indexed. Datasets were recorded at multiple energies, determined from the individual absorption spectra. For each compound and absorption edge, an initial model was obtained by a structure solution with *ShelXT*[8] and then a refinement of positional and anisotropic parameters for all atoms using *olex2.refine*[9] within the *Olex2*[10] crystallographic suite. The initial structural model was then used as an input for serial refinement against the structure factors (.hkl files) obtained at the subsequent energies. This was done using the *Olex2*[10] extension module *SISYPHOS*[11], which guided the serial refinement of anomalous dispersion correctional terms and the extinction correction in an automated fashion employing *olex2.refine*[9]. The output of this process can be found in .csv format containing resulting quality parameters, shift, ADPs, anomalous dispersion values, and more in the

additional supplementing material (ASM1). A .cif is also provided in a zipped folder (ASM2) for each experiment at a different wavelength.

For the cesium uranium titanates, anharmonic displacement parameters were refined for the cesium atoms, and in $Cs_2(UO_2)TiO_4$ half a molecule of water was identified at a chemically plausible position and included in the refinement with a fixed occupancy of 0.5 due to the symmetry constraint. Further information can be found in Table S1 containing the crystallographic information of the starting models.

Table S1: Crystallographic details of the starting crystal structures for the serial refinement of the studied compounds at different wavelengths.

Sample	**$NaUF_5$**	**NaU_2F_9**	**$Cs_2(UO_2)TiO_4$**	**$Cs_2(UO_2)Ti_2O_6$**
X-ray Energy / eV	17,100	17,100	17,000	17,000
Empirical formula	$NaUF_5$	NaU_2F_9	$Cs_2(UO_2)TiO_4 \cdot 0.5\ H_20$	$Cs_2(UO_2)Ti_2O_6$
CCDC Nr.	2337534	2337500	2337547	2337544
Formula weight / g·mol^{-1}	356.011	670.033	1313.421	727.569
Crystal size / mm^{-3}	0.068×0.049×0.030	0.066×0.050×0.043	0.077×0.049×0.039	0.039×0.028×0.025
Temperature / K	294.0(1)	293.9(1)	294.0(1)	294(1)
Space group	Pnma	Pnma	Immm	$P2_1/m$
a / Å	8.70675(4)	8.66316(8)	8.4950(2)	7.51113(10)
b / Å	8.08990(5)	11.1686(1)	13.5769(2)	16.4083(2)
c / Å	10.42316(5)	7.02070(6)	14.5795(4)	7.95961(10)
α, β, γ / °	90	90	90	90, 102.737(1), 90
V / Å^{-3}	734.174(6)	679.29(2)	1681.54(7)	956.84(2)
ρ / g·cm^{-3}	6.442	6.552	5.188	5.051
μ / mm^{-1}	20.494	22.016	18.881	17.441
θ-range / °	6.22 – 67.78	7.00 – 67.76	54.20 – 68.24	5.10 – 68.26
Total reflections	34779	30713	39930	45501
Data/restr./param.	1480/0/76	1355/0/61	1815/0/106	3743/0/187
Completeness / %	100	100	100	100
Resolution / Å	0.65	0.65	0.65	0.65
R_1, $wR_{2,}$ R_{int} (all data) / %	1.12, 3.13	1.43, 3.64	0.96, 2.41	1.38 3.24
GooF	1.047	1.031	1.060	1.038
Largest diff. peak, hole / e·Å^{-3}	0.89,-0.72	1.16,-1.80	0.52, -0.62	1.56, -1.30
$I/\sigma(I)$	107.3	67.6	104.2	66.4
	$-15 \leq h \leq 15$	$-15 \leq h \leq 15$	$-15 \leq h \leq 15$	$-13 \leq h \leq 13$
Indices range	$-14 \leq k \leq 14$	$-20 \leq k \leq 19$	$-24 \leq k \leq 24$	$-27 \leq k \leq 18$
	$-18 \leq l \leq 18$	$-12 \leq l \leq 12$	$-26 \leq l \leq 25$	$-14 \leq l \leq 14$
f', f'' / electrons	-13.7(1), 6.5(2)	-14.6(2), 7.0(4)	-11.09(7), 5.2(1)	U1: -12.02(7) 6.99(9)<br>U2: -11.73(8) 5.99(10)

Labelling scheme of the starting structures of ADR

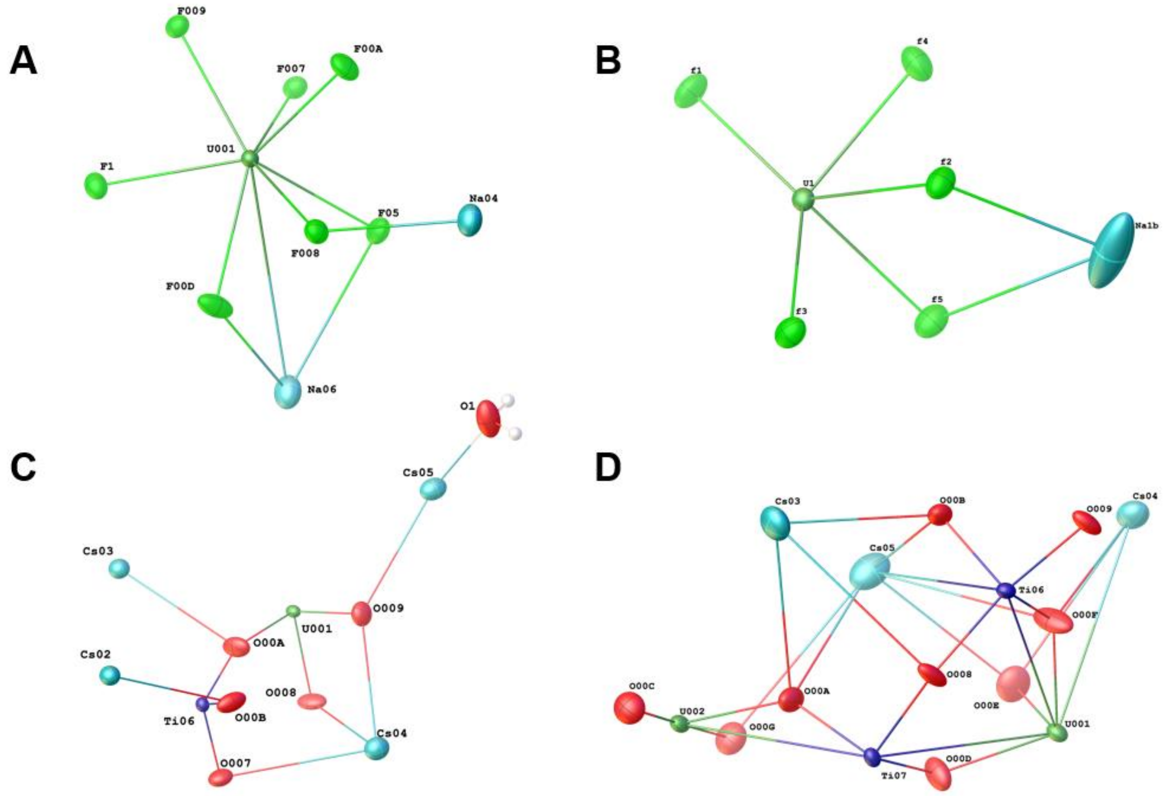

Figure S3: Labelling scheme for $NaUF_5$ (**A**), NaU_2F_9 (**B**), $Cs_2(UO_2)TiO_4$ (**C**), and $Cs_2(UO_2)Ti_2O_6$ (**D**) as given by the respective structure solution program.

Overlay of the starting structure models for ADR and the previously published ones.

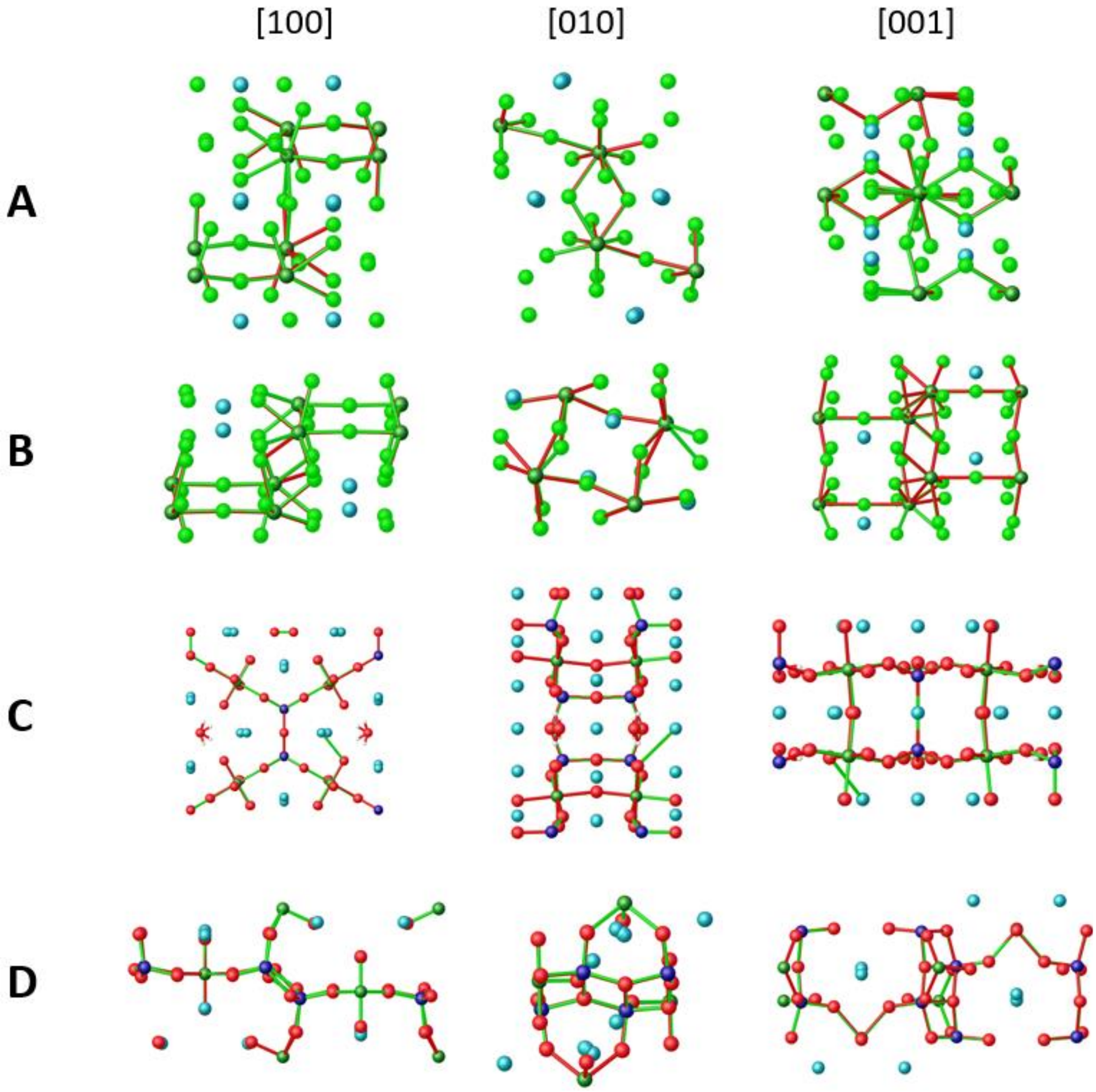

Figure S4: Structural overlay in balls-and-sticks representation of the starting models used in this study and the re-refined published structures of $NaUF_5$ (**A**), NaU_2F_9 (**B**), $Cs_2(UO_2)TiO_4$ (**C**), and $Cs_2(UO_2)Ti_2O_6$ (**D**).

ADR at L_2 and L_1 edges for $NaUF_5$ and $Cs_2(UO_2)TiO_4$

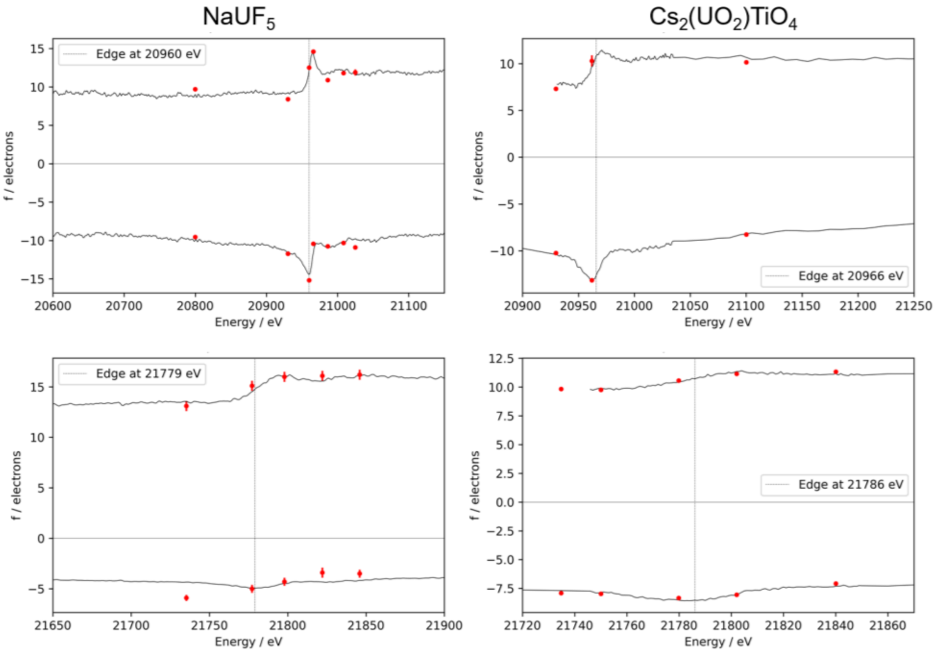

Figure S5: Each plot shows the recorded X-ray absorption spectrum and its Kramers-Kronig transformation for the L_2 (top row) and L_1 edge (bottom) of uranium in two different compounds, together with the freely refined values for f'' and f' from X-ray diffraction data at the different energies. Dispersion parameters are presented with their standard deviations for $NaUF_5$ and $Cs_2(UO_2)TiO_4$. Note that the X-ray absorption spectrum and its transformation were first normalized and then fitted in vertical offset and scaling to the refined anomalous dispersion parameters.

ADR at the U L_3 edges compared to the Brennan & Cowan table

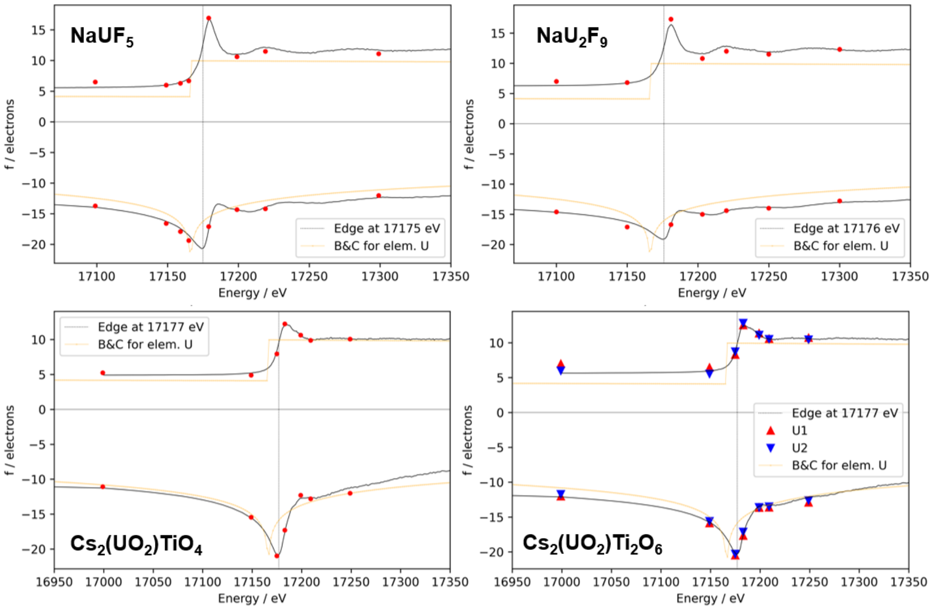

Figure S6: Each plot shows the recorded X-ray absorption spectrum (top) and its Kramers-Kronig transformation (bottom) for the L_3 edge of uranium in four different compounds, together with the freely refined values for f'' (top) and f' (bottom) from X-ray diffraction data at the different energies. Dispersion parameters

are presented with their standard deviations for $NaUF_5$ and $Cs_2(UO_2)TiO_4$. Note that the X-ray absorption spectrum as well as its transformation were first normalized and then fitted in vertical offset as well as scaling to the refined anomalous dispersion parameters.

Table S2: Values and standard deviations for f' and f'' obtained by ADR at various energies around the L_3, L_2, and L_1 absorption edges for $NaUF_5$ and $NaUF_9$.

**$NaUF_5$**		**$NaUF_9$**	
Energy / eV	f', f'' / *e*	Energy / eV	f', f'' / *e*
		L_3	
17,100	-13.7(1), 6.5(2)	17,100	-14.6(2), 7.0(4)
17,150	-16.6(1), 6.0(2)	17,150	-17.1(2), 6.8(3)
17,160	-17.9(1), 6.3(2)	17,181	-16.7(3), 17.3(4)
17,166	-19.4(1), 6.7(1)	17,203	-15.0(3), 10.8(4)
17,180	-17.1(2), 17.0(2)	17,220	-14.4(3), 12.0(4)
17,200	-14.3(2), 10.6(2)	17,250	-14.0(3), 11.5(4)
17,220	-14.3(2), 11.5(2)	17,300	-12.8(3), 12.3(4)
17,300	-12.0(2), 11.1(2)		
		L_2	
20,800	-9.6(1), 9.7(2)	20,800	-8.6(3), 11.4(3)
20,930	-11.7(1), 8.4(3)	20,950	-12.3(2), 10.3(3)
20,960	-15.2(1), 12.5(2)	20,965	-10.5(2), 16.8(3)
20,966	-10.4(2), 14.6(2)	20,988	-9.7(3), 12.7(3)
20,986	-10.8(2), 10.9(2)	21,200	-8.0(3), 14.4(4)
21,008	-10.3(2), 11.8(2)		
21,025	-10.9(3), 11.9(4)		
		L_1	
21,735	-5.9(3), 13.1(5)		
21,777	-5.0(4), 15.1(5)		
21,798	-4.3(4), 16.0(5)		
21,822	-3.4(5), 16.1(5)		
21,846	-3.5(4), 16.2(5)		
21,930	-3.7(4), 15.7(5)		
22,000	-4.0(4), 15.6(5)		

Table S3: Values and standard deviations for f' and f'' obtained by ADR at various energies around the L_3, L_2, and L_1 absorption edges for $Cs_2(UO_2)TiO_4$ and $Cs_2(UO_2)Ti_2O_6$.

$Cs_2(UO_2)TiO_4$		$Cs_2(UO_2)Ti_2O_6$		
Energy / eV	f', f'' / *e*	Energy / eV	f', f'' / *e* (U1)	f', f'' / *e* (U2)
		L_3		
17,000	-11.1(1), 5.2(1)	17,000	-12.0(1), 7.0(1)	-11.7(1), 6.0(1)
17,150	-15.4(1), 4.9(1)	17,150	-15.9(7), 6.5(1)	-15.6(1), 5.5(1)
17,176	-21.0(2), 8.0(1)	17,176	-20.5(1), 8.3(1)	-20.3(1), 8.8(1)
17,184	-17.3(2), 12.2(1)	17,184	-17.7(1), 12.5(6)	-17.1(1), 12.8(1)
17,200	-12.3(3), 10.6(1)	17,200	-13.7(7), 11.4(1)	-13.6(7), 11.1(1)
17,210	-12.8(2), 9.9(1)	17,210	-13.6(1), 10.6(1)	-13.5(1), 10.5(1)
17,250	-12.0(2), 10.1(1)	17,250	-12.9(1), 10.7(1)	-12.6(1), 10.5(1)
		L_2		
20,930	-10.2(1), 7.3(1)	20,930	-10.9(1), 8.5(1)	-10.6(1), 8.01(1)
20,962	-13.2(1), 10.3(6)	20,962	-13.9(1), 10.2(1)	-13.7(1), 10.3(1)
21,100	-8.3(1), 10.2(1)	20,970	-11.2(1), 12.1(1)	-11.2(1), 12.2(1)
		21,100	-8.9(1), 10.9(1)	-8.6(1), 10.7(1)
		L_1		
21,735	-7.9(1), 9.8(1)	21,735	-8.6(1), 10.3(1)	-8.3(1), 10.1(1)
21,750	-8.0(1), 9.8(1)	21,750	-8.7(1), 10.4(1)	-8.4(1), 10.3(1)
21,780	-8.3(1), 10.6(1)	21,780	-9.0(1), 11.1(1)	-9.0(1), 10.8(1)
21,802	-8.1(1), 11.1(1)	21,802	-8.7(1), 11.5(1)	-8.6(1), 11.5(1)
21,840	-7.1(1), 11.3(1)	21,840	-8.0(1), 11.7(1)	-7.9(1), 11.4(1)

Influence of charged atomic form factors on the refinement of anomalous dispersion parameters

For uranium, the International Union for Crystallography (IUCr) has four different sets of atomic form factor parameters in its Tables[12]. These are for the neutral uranium atom used in this study, as well as adaptations for the charged species U^{3+}, U^{4+}, and U^{6+}. The spherical atomic form factor is conventionally approximated by four Gaussian functions and a set of nine parameters in the form of

$$f(|Q|) = \sum_{i=1}^{4} a_i \exp(-b_i(\frac{Q}{4\pi})^2) + c_i$$

Where Q is the scattering vector and *a*, *b*, and *c* are the parameters fitting the Gaussian functions to the Fourier transform of the calculated atomic electron density. Table S4 shows the different parameters and Fig S6 shows the atomic form factors with increasing scattering vector.

Table S4: Fitting parameters of the four different uranium species given in reference [12].

Species	a1	b1	a2	b2	a3	b3	a4	b4	c
U	36.0228	0.5293	23.4128	3.3253	14.9491	16.0927	4.188	100.613	13.3966
U3+	35.5747	0.52048	22.5259	3.12293	12.2165	12.7148	5.37073	26.3394	13.3092
U4+	35.3715	0.516598	22.5326	3.05053	12.0291	12.5723	4.7984	23.4582	13.2671
U6+	34.8509	0.507079	22.7584	2.8903	14.0099	13.1767	1.21457	25.2017	13.1665

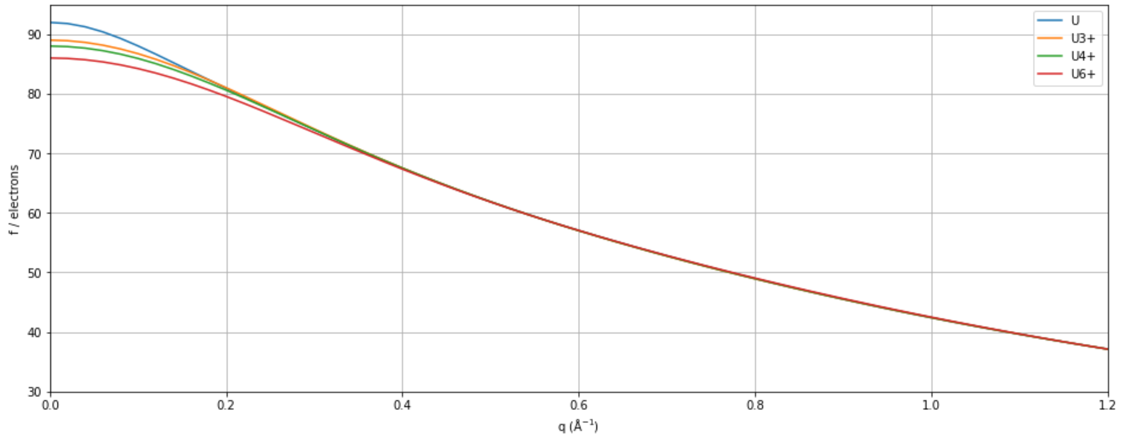

Figure S7: Atomic form factors for neutral and charged uranium species.

Fig. S6 shows, that the difference between the charged species in predominately in the low-resolution region. From about 0.3 Å^{-1}, there is no significant difference between the four species.

To investigate the influence of charged atomic form factors, we took the “most suitable” charged atomic form factor for each of our ADR experiments at the L_3 absorption edge and compared it to the ADR result using the neutral atomic form factor. Fig. S7 shows the side-by-side comparison of the U^{4+} atomic form factor for the fluorides and the U^{6+} form factor for the titanate structures.

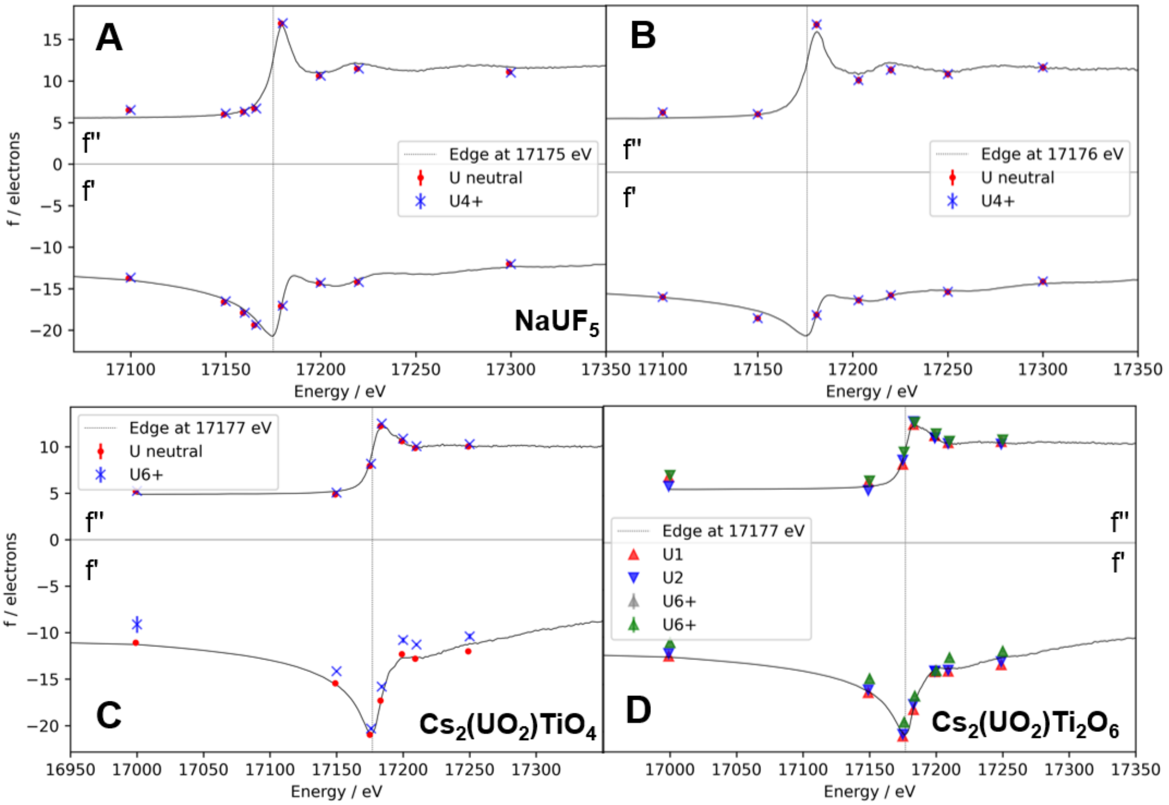

Figure S8: Comparison of ADR using neutral and charged atomic form factors. Figure according to Fig. 2 and 3 in the main text.

Generally, the change of the neutral to the respective charged atomic form factors (using tabulated anomalous dispersion correction values) did not improve the structural quality in terms of quality indicators as well as uranium ADPs (see ASM 3).

No significant difference was observed for the uranium fluorides when neutral and charged (U^{4+}) atomic form factors were used in the ADR procedure. However, for the uranyl titanates, a small difference especially in f' was observed, which is consistent with the higher charge on the metal atom and the direct correction that the real part of the anomalous dispersion correction has on the atomic form factor.

Residual density improvements of the published structures analogous to Figure 4 in the main text

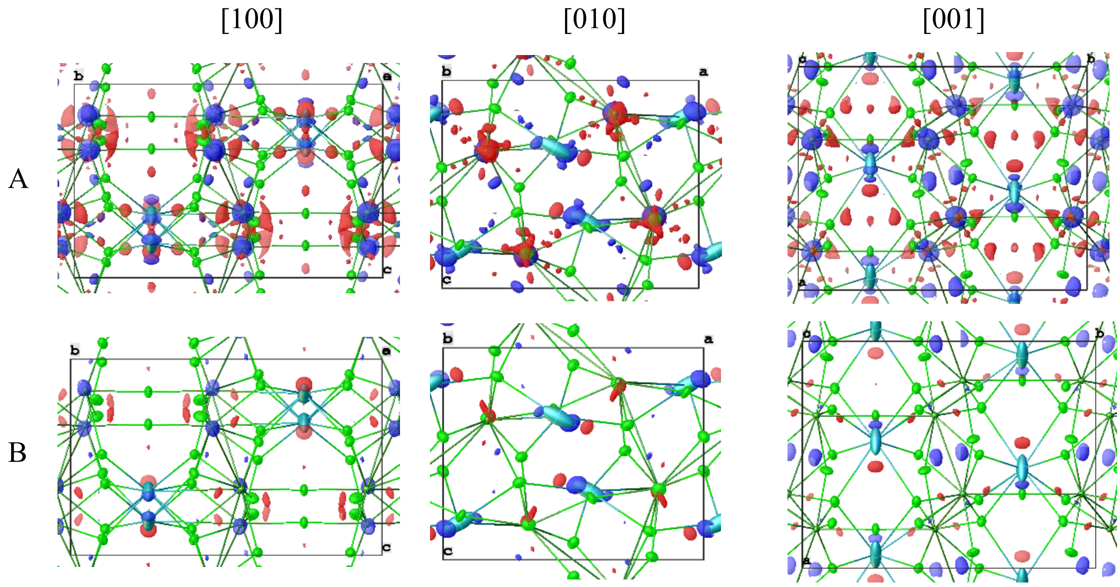

Figure S9: Residual electron density maps at 0.6 *e*/Å^3 iso-level (blue: positive, red: negative) for the published structure of NaU_2F_9 (CCDC 1827660)[1] refined as published using Henke (A) and refined anomalous dispersion values (B) in crystallographic a, b, and c direction. Light green atoms: fluorine, dark green atoms: uranium, cyan atoms: sodium.

[100] [010] [001]

A

B

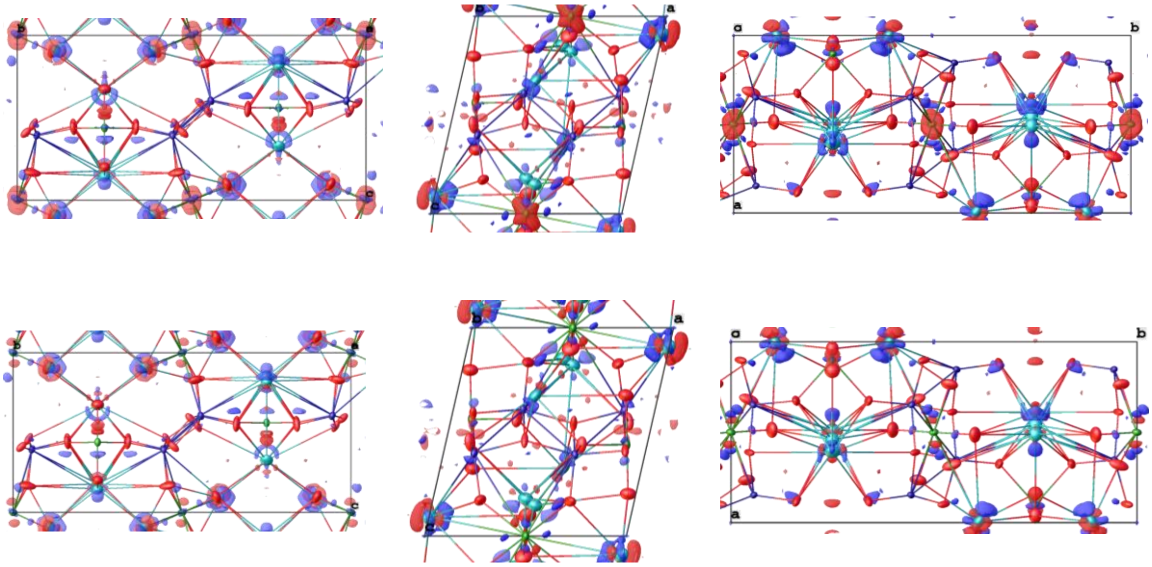

Figure S10: Residual electron density maps at 0.6 *e*/Å^3 iso-level (blue: positive, red: negative) for the published structure of $Cs_2(UO_2)Ti_2O_8$ (CCDC 2033375) refined as published using Henke (A) and refined anomalous dispersion values (B) in crystallographic a, b, and c direction. A newly found water position was also modelled. Red atoms: oxygen, dark green atoms: uranium, dark blue atoms: titanium, cyan atoms: cesium.

[100] [010] [001]

A

B

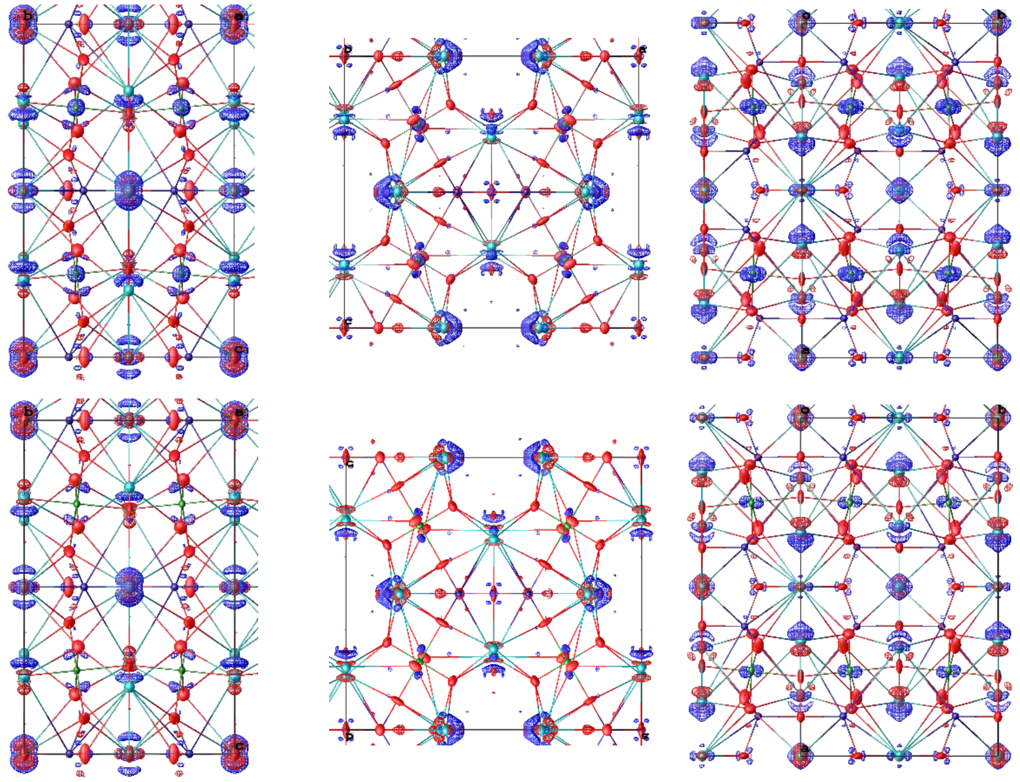

C

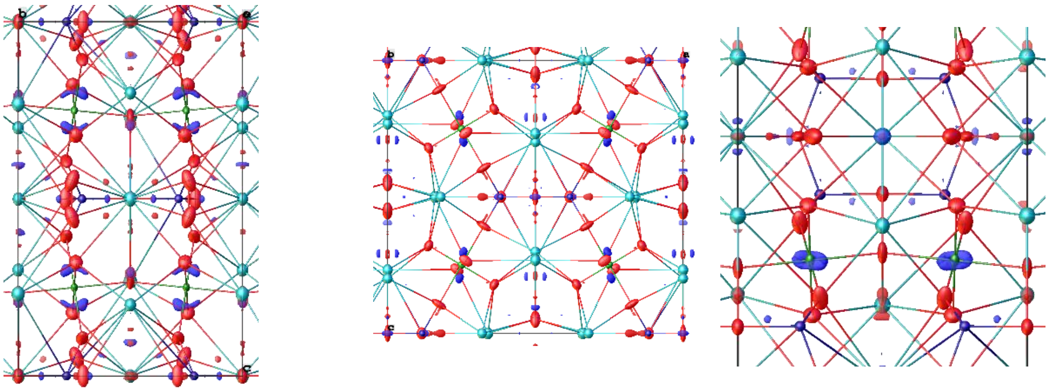

Figure S11: Residual electron density maps at 0.6 *e*/Å^3 iso-level (blue: positive, red: negative) for the published structure of $Cs_2(UO_2)Ti_2O_6$ (CCDC 2033376) refined as published using Henke (A) and refined anomalous dispersion (B) values in crystallographic a, b, and c direction. Red atoms: oxygen, dark green atoms: uranium, dark blue atoms: titanium, cyan atoms: cesium. In C, also anharmonicity and the improved atomic form factors according to Thakkar were used.[13]

Comparison of literature ADR with a different in-house machine

The same crystals subject to this study were remeasured in the University of Regensburg X-ray department to compare the reliability of the values obtained from ADR at in-house machines. The crystals were measured using a Rigaku XtaLAB Synergy-DW rotating anode machine using a HyPix-Arc 150° photon counting detector.

The results are compared in Tab. S5 to the published structures of different crystals of the same material. The resulting .cifs can be found in the additional support material 2 (ADM2, “Synergy-DW”). In general, values for the uraniumfluorides differed more strongly than for the titanates. In the latter case, the deviation was much smaller than even between the different tables for tables for anomalous dispersion correction.

The values for the fluorides differed more significantly in the newly recorded data sets. This could be attributed to the different crystal specimens, which differed more greatly in size for these highly absorbing materials. This trend of a general shift towards higher values for f’ and f” is in accord with the main findings of our study, where a whole series of synchrotron data sets show an offset compared to the theoretical values for isolated atoms.

Table S5: Results of the comparison experiments using Mo Kα radiation at inhouse devices, quality parameters determined at a common resolution of 0.75 Å.

	$NaUF_5$		NaU_2F_9	
	**ADR***	**Mo Kα DW**	**ADR***	**Mo Kα DW**
**R_1,**	1.26,	1.16,	1.33,	0.83,
**wR_2 / %**	2.50	3.57	2.98	1.98
**min., max.**	-0.97,	-1.31,	-0.89,	-1.09,
**peaks / eÅ^{-3}**	0.97	1.00	1.03	1.07
**weights**	0.00,	0.02,	0.01,	0.00,
	1.92	2.83	0.53	3.50
**f',**	-11.9(3),	-12.7(2),	-11.1(3),	-13.6(2),
**f'' / e**	11.8(6)	14.1(3)	9.9(6)	14.7(3)

	$Cs_2(UO_2)TiO_4$		$Cs_2(UO_2)Ti_2O_6$	
	**ADR***	**Mo Kα DW**	**ADR***	**Mo Kα DW**
**R_1,**	1.08,	2.43,	1.08,	1.37,
**wR_2 / %**	2.42	6.70	2.51	2.88
**min., max.**	-0.62, 0.67	-1.75,	-0.94	-1.66,
**peaks / eÅ^{-3}**		2.44	1.53	1.33
**weights**	0.00,	0.04,	0.01,	0.00,
	19.2	15.1	2.39	4.17
**f',**	-10.2(4),	-10.1(2),	-10.5(3) / -11.3(4)	-10.7(1) / -11.0(1)
**f'' / e**	10.5(8)	10.5(2)	10.2(4) / 10.1(5)	11.0(1) / 11.8(1)

* These data sets are re-refined from the published ones[1,2] using olex2.refine[9] and Thakkar IAM[13,14] in Olex2[10]. Different crystals were used in the published structures. For reference, at the Mo Kα wavelength, the tables of Brennen & Cowan[15] (-9.7, 9.7), Henke *et al.*[16] (f': -10.212 *e*, f'': 10.405 *e*) and Sasaki[17] (f': -11.031 *e*, f'': 9.690 *e*) also differ strongly.

References

(1) Klepov, V. V.; Felder, J. B.; zur Loye, H.-C. Synthetic Strategies for the Synthesis of Ternary Uranium(IV) and Thorium(IV) Fluorides. *Inorg. Chem.* **2018**, *57* (9), 5597–5606. https://doi.org/10.1021/acs.inorgchem.8b00570.

(2) Morrison, G.; Christian, M. S.; Besmann, T. M.; zur Loye, H.-C. Flux Growth of Uranyl Titanates: Rare Examples of TiO4 Tetrahedra and TiO5 Square Bipyramids. *J. Phys. Chem. A* **2020**, *124* (45), 9487–9495. https://doi.org/10.1021/acs.jpca.0c08869.

(3) Scheinost, A. C.; Claussner, J.; Exner, J.; Feig, M.; Findeisen, S.; Hennig, C.; Kvashnina, K. O.; Naudet, D.; Prieur, D.; Rossberg, A.; Schmidt, M.; Qiu, C.; Colomp, P.; Cohen, C.; Dettona, E.; Dyadkin, V.; Stumpf, T. ROBL-II at ESRF: A Synchrotron Toolbox for Actinide Research. *J Synchrotron Rad* **2021**, *28* (1), 333–349. https://doi.org/10.1107/S1600577520014265.

(4) Dyadkin, V.; Pattison, P.; Dmitriev, V.; Chernyshov, D. A New Multipurpose Diffractometer PILATUS@SNBL. *J Synchrotron Rad* **2016**, *23* (3), 825–829. https://doi.org/10.1107/S1600577516002411.

(5) Rigaku Oxford Diffraction Ltd. Crysalis PRO, 2019.

(6) Ashiotis, G.; Deschildre, A.; Nawaz, Z.; Wright, J. P.; Karkoulis, D.; Picca, F. E.; Kieffer, J. The Fast Azimuthal Integration Python Library: pyFAI. *J Appl Cryst* **2015**, *48* (2), 510–519. https://doi.org/10.1107/S1600576715004306.

(7) Guzei, I. A.; Bikzhanova, G. A.; Spencer, L. C.; Timofeeva, T. V.; Kinnibrugh, T. L.; Campana, C. F. Polymorphism and History of 2-Dimethylsufuranylidene-1,3-Indanedione (YLID). *Crystal Growth & Design* **2008**, *8* (7), 2411–2418. https://doi.org/10.1021/cg701260p.

(8) Sheldrick, G. M. *SHELXT* – Integrated Space-Group and Crystal-Structure Determination. *Acta Crystallogr A Found Adv* **2015**, *71* (1), 3–8. https://doi.org/10.1107/S2053273314026370.

(9) Bourhis, L. J.; Dolomanov, O. V.; Gildea, R. J.; Howard, J. A. K.; Puschmann, H. The Anatomy of a Comprehensive Constrained, Restrained Refinement Program for the Modern Computing Environment – *Olex2* Dissected. *Acta Crystallogr A* **2015**, *71* (1), 59–75. https://doi.org/10.1107/S2053273314022207.

(10) Dolomanov, O. V.; Bourhis, L. J.; Gildea, R. J.; Howard, J. A. K.; Puschmann, H. *OLEX2* : A Complete Structure Solution, Refinement and Analysis Program. *J Appl Cryst* **2009**, *42* (2), 339–341. https://doi.org/10.1107/S0021889808042726.

(11) FlorianMeurer. FlorianMeurer/Plugin-SISYPHOS, 2023. https://github.com/FlorianMeurer/plugin-SISYPHOS (accessed 2024-02-14).

(12) Brown, P. J.; Fox, A. G.; Maslen, E. N.; O'Keefe, M. A.; Willis, B. T. M. Intensity of Diffracted Intensities. In *International Tables for Crystallography, Online MRW*; John Wiley & Sons, Ltd, 2006; pp 554–595. https://doi.org/10.1107/97809553602060000600.

(13) Kleemiss, F.; Peyerimhoff, N.; Bodensteiner, M. Refinement of X-Ray and Electron Diffraction Crystal Structures Using Analytical Fourier Transforms of Slater-Type Atomic Wavefunctions in Olex2. *J Appl Cryst* **2024**, *57* (1), 161–174. https://doi.org/10.1107/S1600576723010981.

(14) Kleemiss, F.; Dolomanov, O. V.; Bodensteiner, M.; Peyerimhoff, N.; Midgley, L.; Bourhis, L. J.; Genoni, A.; Malaspina, L. A.; Jayatilaka, D.; Spencer, J. L.; White, F.; Grundkötter-Stock, B.; Steinhauer, S.; Lentz, D.; Puschmann, H.; Grabowsky, S. Accurate Crystal Structures and Chemical Properties from NoSpherA2. *Chem. Sci.* **2021**, *12* (5), 1675–1692. https://doi.org/10.1039/D0SC05526C.

(15) Brennan, S.; Cowan, P. L. A Suite of Programs for Calculating X-Ray Absorption, Reflection, and Diffraction Performance for a Variety of Materials at Arbitrary Wavelengths. *Rev. Sci. Instrum.* **1992**, *63* (1), 850–853. https://doi.org/10.1063/1.1142625.

(16) Henke, B. L.; Lee, P.; Tanaka, T. J.; Shimabukuro, R. L.; Fujikawa, B. K. Low-Energy x-Ray Interaction Coefficients: Photoabsorption, Scattering, and Reflection: E = 100–2000 eV Z = 1–94.

Atomic Data and Nuclear Data Tables **1982**, *27* (1), 1–144. https://doi.org/10.1016/0092-640X(82)90002-X.

(17) Sasaki, Satoshi. *Numerical Tables of Anomalous Scattering Factors Calculated by the Cromer and Liberman's Method*; Japan, 1989; p 137.
